# A quality improvement project: documentation of liaison psychiatry patient reviews in the John Radcliffe Hospital, Oxford

**DOI:** 10.1192/bjo.2021.597

**Published:** 2021-06-18

**Authors:** Alice Talks, Susan Shaw, Tomasz Bajorek, Lindsay Carpenter, Anya Topiwala

**Affiliations:** OUH

## Abstract

**Aims:**

Assess how current practice reflects recommendations from the National Confidential Enquiry into Patient Outcome and Death (NCEPOD) Treat as One: bridging the gap between mental and physical healthcare report (January 2017).

Develop template for electronic documentation of liaison psychiatry reviews and implement for trial period.

Re-audit after trial period to assess for change in quality of documentation.

**Background:**

The John Radcliffe Hospital (JR) is a tertiary centre and has a large liaison psychiatry department with 14 consultants. Patient reviews by the liaison team are documented using a blank note type, on an electronic system used by all specialties within the hospital trust. The NCEPOD Treat as One report makes recommendations for the content of documentation of liaison psychiatry reviews which aim to improve communication between specialties.

**Method:**

86 patients referred to liaison psychiatry at the JR in September 2018 were randomly selected. Four liaison psychiatry consultants appraised the quality of documentation of anonymized reviews by consultant colleagues. The audit tool was a questionnaire containing 12 questions developed by the four consultants based on the NCEPOD Treat as One report. Data were collated from these questionnaires. The template for electronic documentation was developed to reflect the report recommendations and after discussion with the liaison psychiatry team. The template has been implemented and is used for all initial patient reviews.

**Result:**

The 12 questions of the audit tool can be divided into two groups: assessment and management. As part of the assessment, the majority of reviews included a primary diagnosis (77.9%) and reason for referral (66.3%). Other aspects of the assessment were documented in the minority of reviews: mental capacity (19.8%), need for DOLS (2.3%), risks (27.9%) and risk management (7%). Regarding the management, the majority of reviews included: clear plan with numbered/bullet points (61.6%), medication changes (51.4%), useful plan (73%) and answered the reason for referral (69.8%). Other aspects of the management were documented in the minority of reviews: each action point assigned (47.7%) and non-medical MDT advice (18.6%).

**Conclusion:**

The main area for improvement in documentation of assessment agreed by the liaison team is risk. The main areas agreed for improvement in documentation of management are medication changes, assigning action points to individuals, and including advice for non-medical MDT members. The next step is re-audit, planned for March 2020.

